# Norm inequalities for functions of matrices

**DOI:** 10.1016/j.heliyon.2024.e30056

**Published:** 2024-04-23

**Authors:** Ahmad Al-Natoor

**Affiliations:** Department of Mathematics, Faculty of Sciences, Isra University, Amman 11622, Jordan

**Keywords:** 15A60, 47A30, 47B15, Spectral norm, Unitarily invariant norm, Positive semidefinite matrix, Matrix, Inequality, Function

## Abstract

In this paper, we prove several spectral norm and unitarily invariant norm inequalities for matrices in which the special cases of our results present some known inequalities. Also, some of our results give interpolating inequalities which are related to well-known recent inequalities.

## 1 Introduction

Let $M_{n}$ denote the space of all square complex matrices of size nxn. The unitarily invariant norm, written as |||⋅|||, is defined on $M_{n}$ and satisfies the property $\left|||U_{1}MU_{2}|||=|||M||\right|$ for $M\in M_{n}$ and for unitaries $U_{1}$ and $U_{2}$. The spectral norm, written as ‖⋅‖, is defined on $M_{n}$ by $\left\|M\right\|=\max_{\left\|x\right\|=1}\left\|Mx\right\|$ for $M\in M_{n}$ and $x\in C^{n}$. The spectral norm is an example of unitarily invariant norms.

The author in [1] proved that if $M,N\in M_{n}$ and α∈[0,1], then

(1.1) $${\left|\left|\left|MN^{⁎}\right|\right|\right|}^{2}\leq \left|\left|\left|\alpha M^{⁎}M+\left(1-\alpha \right)N^{⁎}N\right|\right|\right|\;\left|\left|\left|\left(1-\alpha \right)M^{⁎}M+\alpha N^{⁎}N\right|\right|\right|.$$

Letting $\alpha =\frac{1}{2}$ in inequality (1.1), we have

(1.2) $$\left|\left|\left|MN^{⁎}\right|\right|\right|\leq \frac{1}{2}\left|\left|\left|M^{⁎}M+N^{⁎}N\right|\right|\right|,$$

which was given in [2], while letting α=0 or α=1 in inequality (1.1), we have

(1.3) $${\left|\left|\left|MN^{⁎}\right|\right|\right|}^{2}\leq \left|\left|\left|M^{⁎}M\right|\right|\right|\;\left|\left|\left|N^{⁎}N\right|\right|\right|,$$

which was given in [3]. Specifying inequalities (1.1), (1.2), and (1.3) to the spectral norm, we have

(1.4) $${\left\|MN^{⁎}\right\|}^{2}\leq \left\|\alpha M^{⁎}M+\left(1-\alpha \right)N^{⁎}N\right\|\;\left\|\left(1-\alpha \right)M^{⁎}M+\alpha N^{⁎}N\right\|,$$

(1.5) $$\left\|MN^{⁎}\right\|\leq \frac{1}{2}\left\|M^{⁎}M+N^{⁎}N\right\|,$$

and

(1.6) $${\left\|MN^{⁎}\right\|}^{2}\leq \left\|M^{⁎}M\right\|\left\|N^{⁎}N\right\|.$$

A generalization of inequality (1.2) is given in [4] by

(1.7) $$\left|\left|\left|MSN^{⁎}\right|\right|\right|\leq \frac{\left\|S\right\|}{2}\left|\left|\left|M^{⁎}M+N^{⁎}N\right|\right|\right|$$

for $M,N,S\in M_{n}$, where *S* is positive semidefinite. The authors in [5] proved that

(1.8) $$\left|\left|\left|MSN^{⁎}\right|\right|\right|\leq \frac{\left|\left|\left|S\right|\right|\right|}{2}\left\|M^{⁎}M+N^{⁎}N\right\|$$

for $M,N,S\in M_{n}$, where *S* is positive semidefinite. Note that if we specify inequalities (1.7) and (1.8) to the spectral norm, then they are equivalent and have the form

(1.9) $$\left\|MSN^{⁎}\right\|\leq \frac{\left\|S\right\|}{2}\left\|M^{⁎}M+N^{⁎}N\right\|.$$

It is known (see, e.g., [6, p. 75]) that if $M,N,S\in M_{n}$, then

(1.10) $$\left|\left|\left|MSN^{⁎}\right|\right|\right|\leq \left\|M\right\|\left\|N\right\|\left|\left|\left|S\right|\right|\right|.$$

Recent related results can be found in [7], [8], [9], [10], and [11].

In our paper, we give norm inequalities for functions of matrices. Some of our results generalize some of the above inequalities for special types of functions of matrices.

## 2 Main results

In our analysis, the functions $f_{1}(t),f_{2}(t),g_{1}(t)$, and $g_{2}(t)$ are nonnegative continuous functions on [0,∞) such that $f_{1}(t)f_{2}(t)=t$ and $g_{1}(t)g_{2}(t)=t$. Also, *α* is a real number belongs to the interval [0,1].

Lemma 2.1 *[12]* *Let* $M,N,S\in M_{n}$ *. Then*

$${\left\|MSN^{⁎}\right\|}^{2}\leq \left\|f_{1}(M^{⁎}M)Sg_{2}(N^{⁎}N)\right\|\left\|f_{2}(M^{⁎}M)Sg_{1}(N^{⁎}N)\right\|.$$

In what follows, the matrices *S* and *T* are assumed to be positive semidefinite.

Theorem 2.2 *Let* $M,N,S\in M_{n}$ *. Then*

$${\left\|MSN^{⁎}\right\|}^{2}$$

(2.1) $$\leq {\left\|S\right\|}^{2}\sqrt{\begin{array}{c} \left\|\alpha f_{1}^{2}(M^{⁎}M)+(1-\alpha )g_{2}^{2}(N^{⁎}N)\right\|\left\|(1-\alpha )f_{1}^{2}(M^{⁎}M)+\alpha g_{2}^{2}(N^{⁎}N)\right\|\\ \times \left\|\alpha f_{2}^{2}(M^{⁎}M)+(1-\alpha )g_{1}^{2}(N^{⁎}N)\right\|\left\|(1-\alpha )f_{2}^{2}(M^{⁎}M)+\alpha g_{1}^{2}(N^{⁎}N)\right\| \end{array}.}$$

Proof We have

$$\left\|f_{1}(M^{⁎}M)Sg_{2}(N^{⁎}N)\right\|$$

(2.2) $$=\left\|f_{1}(M^{⁎}M)S^{1/2}S^{1/2}g_{2}(N^{⁎}N)\right\|=\left\|f_{1}(M^{⁎}M)S^{1/2}{\left(g_{2}(N^{⁎}N)S^{1/2}\right)}^{⁎}\right\|\leq \sqrt{\begin{array}{c} \left\|\alpha S^{1/2}f_{1}^{2}(M^{⁎}M)S^{1/2}+(1-\alpha )S^{1/2}g_{2}^{2}(N^{⁎}N)S^{1/2}\right\|\times \\ \left\|(1-\alpha )S^{1/2}f_{1}^{2}(M^{⁎}M)S^{1/2}+\alpha S^{1/2}g_{2}^{2}(N^{⁎}N)S^{1/2}\right\| \end{array}}\;\;\text{(by inequality (1.4))}=\sqrt{\begin{array}{c} \left\|S^{1/2}\left(\alpha f_{1}^{2}(M^{⁎}M)+(1-\alpha )g_{2}^{2}(N^{⁎}N)\right)S^{1/2}\right\|\\ \times \left\|S^{1/2}((1-\alpha )f_{1}^{2}(M^{⁎}M)+\alpha g_{2}^{2}(N^{⁎}N))S^{1/2}\right\| \end{array}}\leq \sqrt{\begin{array}{c} {\left\|S\right\|}^{2}\left\|\alpha f_{1}^{2}(M^{⁎}M)+(1-\alpha )g_{2}^{2}(N^{⁎}N)\right\|\\ \times \left\|(1-\alpha )f_{1}^{2}(M^{⁎}M)+\alpha g_{2}^{2}(N^{⁎}N)\right\| \end{array}}=\left\|S\right\|\sqrt{\begin{array}{c} \left\|\alpha f_{1}^{2}(M^{⁎}M)+(1-\alpha )g_{2}^{2}(N^{⁎}N)\right\|\\ \times \left\|(1-\alpha )f_{1}^{2}(M^{⁎}M)+\alpha g_{2}^{2}(N^{⁎}N)\right\| \end{array}}.$$

Similarly,

$$\left\|f_{2}(M^{⁎}M)Sg_{1}(N^{⁎}N)\right\|$$

(2.3) $$\leq \left\|S\right\|\sqrt{\left\|\alpha f_{2}^{2}(M^{⁎}M)+(1-\alpha )g_{1}^{2}(N^{⁎}N)\right\|\left\|(1-\alpha )f_{2}^{2}(M^{⁎}M)+\alpha g_{1}^{2}(N^{⁎}N)\right\|}.$$

Our result follows from inequalities (2.2) and (2.3) together with Lemma 2.1. □

Letting $\alpha =\frac{1}{2}$ in inequality (2.1), we have

(2.4) $${\left\|MSN^{⁎}\right\|}^{2}\leq \frac{1}{4}{\left\|S\right\|}^{2}\sqrt{\begin{array}{c} \left\|f_{1}^{2}(M^{⁎}M)+g_{2}^{2}(N^{⁎}N)\right\|\left\|f_{1}^{2}(M^{⁎}M)+g_{2}^{2}(N^{⁎}N)\right\|\\ \times \left\|f_{2}^{2}(M^{⁎}M)+g_{1}^{2}(N^{⁎}N)\right\|\left\|f_{2}^{2}(M^{⁎}M)+g_{1}^{2}(N^{⁎}N)\right\| \end{array}},$$

which is a generalization of inequality (1.9) for functions of matrices. This can be observed by letting $f_{1}(t)=f_{2}(t)=g_{1}(t)=g_{2}(t)=t^{1/2}$ in inequality (2.4).

Letting α=1 or α=0 in inequality (2.1), we have

(2.5) $${\left\|MSN^{⁎}\right\|}^{2}\leq {\left\|S\right\|}^{2}\sqrt{\left\|f_{1}^{2}(M^{⁎}M)\right\|\left\|g_{2}^{2}(N^{⁎}N)\right\|\left\|f_{2}^{2}(M^{⁎}M)\right\|\left\|g_{1}^{2}(N^{⁎}N)\right\|},$$

which is a generalization of the spectral norm case of inequality (1.10) for functions of matrices. This can be observed by letting $f_{1}(t)=f_{2}(t)=g_{1}(t)=g_{2}(t)=t^{1/2}$ in inequality (2.5).

Replacing *S* by $S^{1/2}T^{1/2}$ in Lemma 2.1, we have

(2.6) $${\left\|MS^{1/2}T^{1/2}N^{⁎}\right\|}^{2}\leq \left\|f_{1}(M^{⁎}M)S^{1/2}T^{1/2}g_{2}(N^{⁎}N)\right\|\left\|f_{2}(M^{⁎}M)S^{1/2}T^{1/2}g_{1}(N^{⁎}N)\right\|.$$

Corollary 2.3 *Let* $M,N,S,T\in M_{n}$ *. Then*

$${\left\|MS^{1/2}T^{1/2}N^{⁎}\right\|}^{2}$$

$$\leq {\left\|S+T\right\|}^{4}\;\max(\left\|\alpha f_{1}^{2}(M^{⁎}M)\right\|,\left\|(1-\alpha )g_{2}^{2}(N^{⁎}N)\right\|)\times \max(\left\|(1-\alpha )f_{1}^{2}(M^{⁎}M)\right\|,\left\|\alpha g_{2}^{2}(N^{⁎}N)\right\|)\times \max(\left\|\alpha f_{2}^{2}(M^{⁎}M)\right\|,\left\|(1-\alpha )g_{1}^{2}(N^{⁎}N)\right\|)\times \max(\left\|(1-\alpha )f_{2}^{2}(M^{⁎}M)\right\|,\left\|\alpha g_{1}^{2}(N^{⁎}N)\right\|).$$

*In particular, if* $\alpha =\frac{1}{2}$ *, we have*

$${\left\|MS^{1/2}T^{1/2}N^{⁎}\right\|}^{2}\leq \frac{1}{16}{\left\|S+T\right\|}^{4}\;{\left(\max(\left\|f_{1}^{2}(M^{⁎}M)\right\|,\left\|g_{2}^{2}(N^{⁎}N)\right\|)\right)}^{2}\times {\left(\max(\left\|f_{2}^{2}(M^{⁎}M)\right\|,\left\|g_{1}^{2}(N^{⁎}N)\right\|)\right)}^{2}.$$

Proof We have

$$\left\|f_{1}(M^{⁎}M)S^{1/2}T^{1/2}g_{2}(N^{⁎}N)\right\|$$

(2.7) $$=\left\|f_{1}(M^{⁎}M)S^{1/2}{\left(g_{2}(N^{⁎}N)T^{1/2}\right)}^{⁎}\right\|\leq \left\|\alpha S^{1/2}f_{1}^{2}(M^{⁎}M)S^{1/2}+(1-\alpha )T^{1/2}g_{2}^{2}(N^{⁎}N)T^{1/2}\right\|\times \left\|(1-\alpha )S^{1/2}f_{1}^{2}(M^{⁎}M)S^{1/2}+\alpha T^{1/2}g_{2}^{2}(N^{⁎}N)T^{1/2}\right\|\;\text{(by inequality (1.4))}=\left\|\left[\begin{array}{cc} S^{1/2} & T^{1/2}\\ 0 & 0 \end{array}\right]\left[\begin{array}{cc} \alpha f_{1}^{2}(M^{⁎}M) & 0\\ 0 & \left(1-\alpha )g_{2}^{2}(N^{⁎}N\right) \end{array}\right]\left[\begin{array}{cc} S^{1/2} & 0\\ T^{1/2} & 0 \end{array}\right]\right\|\times \left\|\left[\begin{array}{cc} S^{1/2} & T^{1/2}\\ 0 & 0 \end{array}\right]\left[\begin{array}{cc} \left(1-\alpha )f_{1}^{2}(M^{⁎}M\right) & 0\\ 0 & \alpha g_{2}^{2}(N^{⁎}N) \end{array}\right]\left[\begin{array}{cc} S^{1/2} & 0\\ T^{1/2} & 0 \end{array}\right]\right\|\leq \left\|\left[\begin{array}{cc} S^{1/2} & T^{1/2}\\ 0 & 0 \end{array}\right]\right\|\left\|\left[\begin{array}{cc} S^{1/2} & 0\\ T^{1/2} & 0 \end{array}\right]\right\|\left\|\left[\begin{array}{cc} \alpha f_{1}^{2}(M^{⁎}M) & 0\\ 0 & \left(1-\alpha )g_{2}^{2}(N^{⁎}N\right) \end{array}\right]\right\|\times \left\|\left[\begin{array}{cc} S^{1/2} & T^{1/2}\\ 0 & 0 \end{array}\right]\right\|\left\|\left[\begin{array}{cc} S^{1/2} & 0\\ T^{1/2} & 0 \end{array}\right]\right\|\left\|\left[\begin{array}{cc} \left(1-\alpha )f_{1}^{2}(M^{⁎}M\right) & 0\\ 0 & \alpha g_{2}^{2}(N^{⁎}N) \end{array}\right]\right\|\;\text{(by the submultiplicativity of the spectral norm)}={\left\|\left[\begin{array}{cc} S^{1/2} & 0\\ T^{1/2} & 0 \end{array}\right]\right\|}^{2}\left\|\left[\begin{array}{cc} \alpha f_{1}^{2}(M^{⁎}M) & 0\\ 0 & \left(1-\alpha )g_{2}^{2}(N^{⁎}N\right) \end{array}\right]\right\|\times {\left\|\left[\begin{array}{cc} S^{1/2} & 0\\ T^{1/2} & 0 \end{array}\right]\right\|}^{2}\left\|\left[\begin{array}{cc} \left(1-\alpha )f_{1}^{2}(M^{⁎}M\right) & 0\\ 0 & \alpha g_{2}^{2}(N^{⁎}N) \end{array}\right]\right\|=\left\|\left[\begin{array}{cc} S^{1/2} & T^{1/2}\\ 0 & 0 \end{array}\right]\left[\begin{array}{cc} S^{1/2} & 0\\ T^{1/2} & 0 \end{array}\right]\right\|\left\|\left[\begin{array}{cc} \alpha f_{1}^{2}(M^{⁎}M) & 0\\ 0 & \left(1-\alpha )g_{2}^{2}(N^{⁎}N\right) \end{array}\right]\right\|\times \left\|\left[\begin{array}{cc} S^{1/2} & T^{1/2}\\ 0 & 0 \end{array}\right]\left[\begin{array}{cc} S^{1/2} & 0\\ T^{1/2} & 0 \end{array}\right]\right\|\left\|\left[\begin{array}{cc} \left(1-\alpha )f_{1}^{2}(M^{⁎}M\right) & 0\\ 0 & \alpha g_{2}^{2}(N^{⁎}N) \end{array}\right]\right\|={\left\|S+T\right\|}^{2}\;\max(\left\|\alpha f_{1}^{2}(M^{⁎}M)\right\|,\left\|(1-\alpha )g_{2}^{2}(N^{⁎}N)\right\|)\times \max(\left\|(1-\alpha )f_{1}^{2}(M^{⁎}M)\right\|,\left\|\alpha g_{2}^{2}(N^{⁎}N)\right\|).$$

Similarly, one can get

$$\left\|f_{2}(M^{⁎}M)S^{1/2}T^{1/2}g_{1}(N^{⁎}N)\right\|$$

(2.8) $$\leq {\left\|S+T\right\|}^{2}\;\max(\left\|\alpha f_{2}^{2}(M^{⁎}M)\right\|,\left\|(1-\alpha )g_{1}^{2}(N^{⁎}N)\right\|)\times \max(\left\|(1-\alpha )f_{2}^{2}(M^{⁎}M)\right\|,\left\|\alpha g_{1}^{2}(N^{⁎}N)\right\|).$$

From inequalities (2.6), (2.7), and (2.8), we obtained the desired result. □

Corollary 2.4 *Let* $M,N,S,T\in M_{n}$ *. Then*

$${\left\|MS^{1/2}T^{1/2}N^{⁎}\right\|}^{2}$$

$$\leq {\left\|\alpha S+(1-\alpha )T\right\|}^{2}{\left\|(1-\alpha )S+\alpha T\right\|}^{2}{\left(\max(\left\|f_{1}^{2}(M^{⁎}M)\right\|,\left\|g_{2}^{2}(N^{⁎}N)\right\|)\right)}^{2}\times {\left(\max(\left\|f_{2}^{2}(M^{⁎}M)\right\|,\left\|g_{1}^{2}(N^{⁎}N)\right\|)\right)}^{2}.$$

Proof We have

$$\left\|f_{1}(M^{⁎}M)S^{1/2}T^{1/2}g_{2}(N^{⁎}N)\right\|$$

(2.9) $$=\left\|f_{1}(M^{⁎}M)S^{1/2}{\left(g_{2}(N^{⁎}N)T^{1/2}\right)}^{⁎}\right\|\leq \left\|\alpha S^{1/2}f_{1}^{2}(M^{⁎}M)S^{1/2}+(1-\alpha )T^{1/2}g_{2}^{2}(N^{⁎}N)T^{1/2}\right\|\times \left\|(1-\alpha )S^{1/2}f_{1}^{2}(M^{⁎}M)S^{1/2}+\alpha T^{1/2}g_{2}^{2}(N^{⁎}N)T^{1/2}\right\|\;\text{(by inequality (1.4))}=\left\|\left[\begin{array}{cc} \sqrt{\alpha }S^{1/2} & \sqrt{1-\alpha }T^{1/2}\\ 0 & 0 \end{array}\right]\left[\begin{array}{cc} f_{1}^{2}(M^{⁎}M) & 0\\ 0 & g_{2}^{2}(N^{⁎}N) \end{array}\right]\left[\begin{array}{cc} \sqrt{\alpha }S^{1/2} & 0\\ \sqrt{1-\alpha }T^{1/2} & 0 \end{array}\right]\right\|\times \left\|\left[\begin{array}{cc} \sqrt{1-\alpha }S^{1/2} & \sqrt{\alpha }T^{1/2}\\ 0 & 0 \end{array}\right]\left[\begin{array}{cc} f_{1}^{2}(M^{⁎}M) & 0\\ 0 & g_{2}^{2}(N^{⁎}N) \end{array}\right]\left[\begin{array}{cc} \sqrt{1-\alpha }S^{1/2} & 0\\ \sqrt{\alpha }T^{1/2} & 0 \end{array}\right]\right\|\leq \left\|\left[\begin{array}{cc} \sqrt{\alpha }S^{1/2} & \sqrt{1-\alpha }T^{1/2}\\ 0 & 0 \end{array}\right]\right\|\left\|\left[\begin{array}{cc} \sqrt{\alpha }S^{1/2} & 0\\ \sqrt{1-\alpha }T^{1/2} & 0 \end{array}\right]\right\|\left\|\left[\begin{array}{cc} f_{1}^{2}(M^{⁎}M) & 0\\ 0 & g_{2}^{2}(N^{⁎}N) \end{array}\right]\right\|\times \left\|\left[\begin{array}{cc} \sqrt{1-\alpha }S^{1/2} & \sqrt{\alpha }T^{1/2}\\ 0 & 0 \end{array}\right]\right\|\left\|\left[\begin{array}{cc} \sqrt{1-\alpha }S^{1/2} & 0\\ \sqrt{\alpha }T^{1/2} & 0 \end{array}\right]\right\|\left\|\left[\begin{array}{cc} f_{1}^{2}(M^{⁎}M) & 0\\ 0 & g_{2}^{2}(N^{⁎}N) \end{array}\right]\right\|\;\text{(by the submultiplicativity of the spectral norm)}={\left\|\left[\begin{array}{cc} \sqrt{\alpha }S^{1/2} & 0\\ \sqrt{1-\alpha }T^{1/2} & 0 \end{array}\right]\right\|}^{2}\left\|\left[\begin{array}{cc} f_{1}^{2}(M^{⁎}M) & 0\\ 0 & g_{2}^{2}(N^{⁎}N) \end{array}\right]\right\|\times {\left\|\left[\begin{array}{cc} \sqrt{1-\alpha }S^{1/2} & 0\\ \sqrt{\alpha }T^{1/2} & 0 \end{array}\right]\right\|}^{2}\left\|\left[\begin{array}{cc} f_{1}^{2}(M^{⁎}M) & 0\\ 0 & g_{2}^{2}(N^{⁎}N) \end{array}\right]\right\|=\left\|\left[\begin{array}{cc} \sqrt{\alpha }S^{1/2} & \sqrt{1-\alpha }T^{1/2}\\ 0 & 0 \end{array}\right]\left[\begin{array}{cc} \sqrt{\alpha }S^{1/2} & 0\\ \sqrt{1-\alpha }T^{1/2} & 0 \end{array}\right]\right\|\left\|\left[\begin{array}{cc} f_{1}^{2}(M^{⁎}M) & 0\\ 0 & g_{2}^{2}(N^{⁎}N) \end{array}\right]\right\|\times \left\|\left[\begin{array}{cc} \sqrt{1-\alpha }S^{1/2} & \sqrt{\alpha }T^{1/2}\\ 0 & 0 \end{array}\right]\left[\begin{array}{cc} \sqrt{1-\alpha }S^{1/2} & 0\\ \sqrt{\alpha }T^{1/2} & 0 \end{array}\right]\right\|\left\|\left[\begin{array}{cc} f_{1}^{2}(M^{⁎}M) & 0\\ 0 & g_{2}^{2}(N^{⁎}N) \end{array}\right]\right\|=\left\|\alpha S+(1-\alpha )T\right\|\left\|(1-\alpha )S+\alpha T\right\|{\left(\max(\left\|f_{1}^{2}(M^{⁎}M)\right\|,\left\|g_{2}^{2}(N^{⁎}N)\right\|)\right)}^{2}.$$

Similarly, one can get

$$\left\|f_{2}(M^{⁎}M)S^{1/2}T^{1/2}g_{1}(N^{⁎}N)\right\|$$

(2.10) $$\leq \left\|\alpha S+(1-\alpha )T\right\|\left\|(1-\alpha )S+\alpha T\right\|\;{\left(\max(\left\|f_{2}^{2}(M^{⁎}M)\right\|,\left\|g_{1}^{2}(N^{⁎}N)\right\|)\right)}^{2}.$$

From inequalities (2.6), (2.9) and (2.10), we obtained the desired result. □

Lemma 2.5 *[5]* *Let* $M,N,S\in M_{n}$ *. Then*

$$\left|\left|\left|MSN^{⁎}\right|\right|\right|\leq \frac{1}{2}\left\|\frac{M^{⁎}M}{{\left\|M\right\|}^{2}}+\frac{N^{⁎}N}{{\left\|N\right\|}^{2}}\right\|\left\|M\right\|\left\|N\right\|\left|\left|\left|S\right|\right|\right|.$$

*In particular, if* |||⋅|||=‖⋅‖ *, then*

$$\left\|MSN^{⁎}\right\|\leq \frac{1}{2}\left\|\frac{M^{⁎}M}{{\left\|M\right\|}^{2}}+\frac{N^{⁎}N}{{\left\|N\right\|}^{2}}\right\|\left\|M\right\|\left\|N\right\|\left\|S\right\|.$$

Corollary 2.6 *Let* $M,N,S\in M_{n}$ *. Then*

$${\left\|MSN^{⁎}\right\|}^{2}$$

(2.11) $$\leq \frac{{\left\|S\right\|}^{2}}{4}\left\|\frac{f_{1}^{2}(M^{⁎}M)}{{\left\|f_{1}(M^{⁎}M)\right\|}^{2}}+\frac{g_{2}^{2}(N^{⁎}N)}{{\left\|g_{2}(N^{⁎}N)\right\|}^{2}}\right\|\left\|f_{1}(M^{⁎}M)\right\|\left\|g_{2}(N^{⁎}N)\right\|\times \left\|\frac{f_{2}^{2}(M^{⁎}M)}{{\left\|f_{2}(M^{⁎}M)\right\|}^{2}}+\frac{g_{1}^{2}(N^{⁎}N)}{{\left\|g_{1}(N^{⁎}N)\right\|}^{2}}\right\|\left\|f_{2}(M^{⁎}M)\right\|\left\|g_{1}(N^{⁎}N)\right\|.$$

Proof We have

$$\left\|f_{1}(M^{⁎}M)Sg_{2}(N^{⁎}N)\right\|$$

(2.12) $$\leq \frac{1}{2}\left\|\frac{f_{1}^{2}(M^{⁎}M)}{{\left\|f_{1}(M^{⁎}M)\right\|}^{2}}+\frac{g_{2}^{2}(N^{⁎}N)}{{\left\|g_{2}(N^{⁎}N)\right\|}^{2}}\right\|\left\|f_{1}(M^{⁎}M)\right\|\left\|g_{2}(N^{⁎}N)\right\|\left\|S\right\|\;\text{(by Lemma 2.5).}$$

Similarly,

$$\left\|f_{2}(M^{⁎}M)Sg_{1}(N^{⁎}N)\right\|$$

(2.13) $$\leq \frac{1}{2}\left\|\frac{f_{2}^{2}(M^{⁎}M)}{{\left\|f_{2}(M^{⁎}M)\right\|}^{2}}+\frac{g_{1}^{2}(N^{⁎}N)}{{\left\|g_{1}(N^{⁎}N)\right\|}^{2}}\right\|\left\|f_{2}(M^{⁎}M)\right\|\left\|g_{1}(N^{⁎}N)\right\|\left\|S\right\|.$$

Our result follows from inequalities (2.12) and (2.13) together with Lemma 2.1. □

Remark 2.7 The inequality (2.11) gives a refinement and a generalization of inequality (1.10) for the case of spectral norm. This can be observed as follows:

$${\left\|MSN^{⁎}\right\|}^{2}$$

$$\leq \frac{{\left\|S\right\|}^{2}}{4}\left\|\frac{f_{1}^{2}(M^{⁎}M)}{{\left\|f_{1}(M^{⁎}M)\right\|}^{2}}+\frac{g_{2}^{2}(N^{⁎}N)}{{\left\|g_{2}(N^{⁎}N)\right\|}^{2}}\right\|\left\|f_{1}(M^{⁎}M)\right\|\left\|g_{2}(N^{⁎}N)\right\|\times \left\|\frac{f_{2}^{2}(M^{⁎}M)}{{\left\|f_{2}(M^{⁎}M)\right\|}^{2}}+\frac{g_{1}^{2}(N^{⁎}N)}{{\left\|g_{1}(N^{⁎}N)\right\|}^{2}}\right\|\left\|f_{2}(M^{⁎}M)\right\|\left\|g_{1}(N^{⁎}N)\right\|\leq \frac{{\left\|S\right\|}^{2}}{4}\left(\left\|\frac{f_{1}^{2}(M^{⁎}M)}{{\left\|f_{1}(M^{⁎}M)\right\|}^{2}}\right\|+\left\|\frac{g_{2}^{2}(N^{⁎}N)}{{\left\|g_{2}(N^{⁎}N)\right\|}^{2}}\right\|\right)\left\|f_{1}(M^{⁎}M)\right\|\left\|g_{2}(N^{⁎}N)\right\|\times \left(\left\|\frac{f_{2}^{2}(M^{⁎}M)}{{\left\|f_{2}(M^{⁎}M)\right\|}^{2}}\right\|+\left\|\frac{g_{1}^{2}(N^{⁎}N)}{{\left\|g_{1}(N^{⁎}N)\right\|}^{2}}\right\|\right)\left\|f_{2}(M^{⁎}M)\right\|\left\|g_{1}(N^{⁎}N)\right\|\;\text{(by the triangle inequality)}={\left\|S\right\|}^{2}\left\|f_{1}(M^{⁎}M)\right\|\left\|g_{2}(N^{⁎}N)\right\|\left\|f_{2}(M^{⁎}M)\right\|\left\|g_{1}(N^{⁎}N)\right\|.$$

So,

$$\left\|MSN^{⁎}\right\|\leq \left\|S\right\|\sqrt{\left\|f_{1}(M^{⁎}M)\right\|\left\|g_{2}(N^{⁎}N)\right\|\left\|f_{2}(M^{⁎}M)\right\|\left\|g_{1}(N^{⁎}N)\right\|},$$

which is a general version of inequality (1.10) for the case of spectral norm. This can be observed by letting $f_{1}(t)=g_{1}(t)=$ $f_{2}(t)=$ $g_{2}(t)=t^{1/2}$.

In what follows, by the symbol |M|, we mean the absolute value of the matrix $M\in M_{n}$, which is defined by $\left|M\right|={\left(M^{⁎}M\right)}^{1/2}$.

Lemma 2.8 *[6, p. 253]* *Let* $M,N\in M_{n}$ *be such that MN is normal. Then*

|||MN|||≤|||NM|||.

Lemma 2.9 *[12]* *Let* $M,N\in M_{n}$ *. Then*

$${\left|\left|\left|MN^{⁎}\right|\right|\right|}^{2}\leq \left|\left|\left|{\left(M^{⁎}M\right)}^{\alpha }{\left(N^{⁎}N\right)}^{1-\alpha }\right|\right|\right|\;\left|\left|\left|{\left(M^{⁎}M\right)}^{1-\alpha }{\left(N^{⁎}N\right)}^{\alpha }\right|\right|\right|.$$

Theorem 2.10 *Let* $M,N\in M_{n}$ *be such that* ${\left|M\right|}^{2}{\left|N\right|}^{2}={\left|N\right|}^{2}{\left|M\right|}^{2}$ *. Then*

$$\left|\left|\left|\;{\left|MN^{⁎}\right|}^{2}\right|\right|\right|$$

(2.14) $$\leq \sqrt{\begin{array}{c} \left|\left|\left|\alpha g_{2}^{2}(M^{⁎}M)+(1-\alpha )f_{1}^{2}(N^{⁎}N)\right|\right|\right|\;\left|\left|\left|(1-\alpha )g_{2}^{2}(M^{⁎}M)+\alpha f_{1}^{2}(N^{⁎}N)\right|\right|\right|\\ \times \left|\left|\left|\alpha f_{2}^{2}(N^{⁎}N)+(1-\alpha )g_{1}^{2}(M^{⁎}M)\right|\right|\right|\;\left|\left|\left|\left(1-\alpha \right)f_{2}^{2}(N^{⁎}N)+\alpha g_{1}^{2}(M^{⁎}M)\right|\right|\right| \end{array}}$$

*and*

(2.15) $${\left\|MN^{⁎}\right\|}^{2}\leq \sqrt{\begin{array}{c} \left\|\alpha g_{2}^{2}(M^{⁎}M)+(1-\alpha )f_{1}^{2}(N^{⁎}N)\right\|\;\left\|(1-\alpha )g_{2}^{2}(M^{⁎}M)+\alpha f_{1}^{2}(N^{⁎}N)\right\|\\ \times \left\|\alpha f_{2}^{2}(N^{⁎}N)+(1-\alpha )g_{1}^{2}(M^{⁎}M)\right\|\left\|\left(1-\alpha \right)f_{2}^{2}(N^{⁎}N)+\alpha g_{1}^{2}(M^{⁎}M)\right\| \end{array}}.$$

Proof Since the matrix $NM^{⁎}MN^{⁎}$ is positive semidefinite (and hence normal), we have

(2.16) $$\left|\left|\left|\;{\left|MN^{⁎}\right|}^{2}\right|\right|\right|=\left|\left|\left|\;NM^{⁎}MN^{⁎}\right|\right|\right|\leq \left|\left|\left|N^{⁎}NM^{⁎}M\right|\right|\right|\;\text{(by}\;\text{Lemma 2.8}\text{)}=\left|\left|\left|f_{1}(N^{⁎}N)f_{2}(N^{⁎}N)g_{1}(M^{⁎}M)g_{2}(M^{⁎}M)\right|\right|\right|.$$

Now, since $f_{1}(N^{⁎}N)f_{2}(N^{⁎}N)g_{1}(M^{⁎}M)g_{2}(M^{⁎}M)=N^{⁎}NM^{⁎}M$, which is a positive semidefinite matrix (and hence normal) under the condition ${\left|M\right|}^{2}{\left|N\right|}^{2}={\left|N\right|}^{2}{\left|M\right|}^{2}$, then

$$\left|\left|\left|f_{1}(N^{⁎}N)f_{2}(N^{⁎}N)g_{1}(M^{⁎}M)g_{2}(M^{⁎}M)\right|\right|\right|$$

(2.17) $$\leq \left|\left|\left|g_{2}(M^{⁎}M)f_{1}(N^{⁎}N)f_{2}(N^{⁎}N)g_{1}(M^{⁎}M)\right|\right|\right|\;\text{(by Lemma 2.8)}\leq \left|\left|\left|g_{2}(M^{⁎}M)f_{1}(N^{⁎}N)\right|\right|\right|\;\left|\left|\left|f_{2}(N^{⁎}N)g_{1}(M^{⁎}M)\right|\right|\right|\leq \sqrt{\begin{array}{c} \left|\left|\left|{\left(g_{2}^{2}(M^{⁎}M)\right)}^{\alpha }{\left(f_{1}^{2}(N^{⁎}N)\right)}^{1-\alpha }\right|\right|\right|\;\left|\left|\left|{\left(g_{2}^{2}(M^{⁎}M)\right)}^{1-\alpha }{\left(f_{1}^{2}(N^{⁎}N)\right)}^{\alpha }\right|\right|\right|\;\\ \times \left|\left|\left|{\left(f_{2}^{2}(N^{⁎}N)\right)}^{\alpha }{\left(g_{1}^{2}(M^{⁎}M)\right)}^{1-\alpha }\right|\right|\right|\;\left|\left|\left|{\left(f_{2}^{2}(N^{⁎}N)\right)}^{1-\alpha }{\left(g_{1}^{2}(M^{⁎}M)\right)}^{\alpha }\right|\right|\right| \end{array}}$$

(2.18) $$\;\text{(by Lemma 2.9)}\leq \sqrt{\begin{array}{c} \left|\left|\left|\alpha g_{2}^{2}(M^{⁎}M)+(1-\alpha )f_{1}^{2}(N^{⁎}N)\right|\right|\right|\;\left|\left|\left|(1-\alpha )g_{2}^{2}(M^{⁎}M)+\alpha f_{1}^{2}(N^{⁎}N)\right|\right|\right|\\ \times \left|\left|\left|\alpha f_{2}^{2}(N^{⁎}N)+(1-\alpha )g_{1}^{2}(M^{⁎}M)\right|\right|\right|\;\left|\left|\left|(1-\alpha )f_{2}^{2}(N^{⁎}N)+\alpha g_{1}^{2}(M^{⁎}M)\right|\right|\right| \end{array}}\;\;\text{(by the young inequality).}$$

Now, the inequality (2.14) follows from inequalities (2.16) and (2.18). The inequality (2.15) follows by specifying inequality (2.14) to the spectral norm and using the fact that $\left\|{\left|W\right|}^{2}\right\|={\left\|W\right\|}^{2}$ for any $W\in M_{n}$. □

Letting $\alpha =\frac{1}{2}$ in inequality (2.15), we have

(2.19) $$\left\|MN^{⁎}\right\|\leq \frac{1}{2}\sqrt{\left\|g_{2}^{2}(M^{⁎}M)+f_{1}^{2}(N^{⁎}N)\right\|\;\left\|f_{2}^{2}(N^{⁎}N)+g_{1}^{2}(M^{⁎}M)\right\|}.$$

The inequality (2.19) is a generalization of inequality (1.5) for the special case when ${\left|M\right|}^{2}{\left|N\right|}^{2}={\left|N\right|}^{2}{\left|M\right|}^{2}$. To see this, let $f_{1}(t)=f_{2}(t)=g_{1}(t)=g_{2}(t)=t^{1/2}$ in (2.19).

Letting α=1 or α=0, in inequality (2.15), we have

(2.20) $${\left\|MN^{⁎}\right\|}^{2}\leq \sqrt{\left\|g_{2}^{2}(M^{⁎}M)\right\|\;\left\|f_{1}^{2}(N^{⁎}N)\right\|\left\|f_{2}^{2}(N^{⁎}N)\right\|\left\|g_{1}^{2}(M^{⁎}M)\right\|},$$

which is a generalization of inequality (1.6) for the special case when ${\left|M\right|}^{2}{\left|N\right|}^{2}={\left|N\right|}^{2}{\left|M\right|}^{2}$. To see this, let $f_{1}(t)=f_{2}(t)=g_{1}(t)=g_{2}(t)=t^{1/2}$ in (2.20).

Corollary 2.11 *Let* $M,N\in M_{n}$ *be such that* ${\left|M\right|}^{2}{\left|N\right|}^{2}={\left|N\right|}^{2}{\left|M\right|}^{2}$ *. Then*

$$\left|\left|\left|\;{\left|MN^{⁎}\right|}^{2}\right|\right|\right|\leq \frac{1}{4}\left|\left|\left|g_{2}^{2}(M^{⁎}M)+f_{1}^{2}(N^{⁎}N)\right|\right|\right|\;\left|\left|\left|f_{2}^{2}(N^{⁎}N)+g_{1}^{2}(M^{⁎}M)\right|\right|\right|.$$

Proof By the inequalities (2.16) and (2.17), we have

$$\left|\left|\left|\;{\left|MN^{⁎}\right|}^{2}\right|\right|\right|\leq \left|\left|\left|g_{2}(M^{⁎}M)f_{1}(N^{⁎}N)\right|\right|\right|\;\left|\left|\left|f_{2}(N^{⁎}N)g_{1}(M^{⁎}M)\right|\right|\right|\leq \frac{1}{4}\left|\left|\left|g_{2}^{2}(M^{⁎}M)+f_{1}^{2}(N^{⁎}N)\right|\right|\right|\;\left|\left|\left|f_{2}^{2}(N^{⁎}N)+g_{1}^{2}(M^{⁎}M)\right|\right|\right|\;\text{(by inequality (1.2)),}$$

as required. □

Lemma 2.12 *[13]* *Let* $S,T\in M_{n}$ *. Then*

$$\left|\left|\left|ST\right|\right|\right|\leq \frac{1}{4}\left|\left|\left|{\left(S+T\right)}^{2}\right|\right|\right|.$$

Corollary 2.13 *Let* $M,N\in M_{n}$ *be such that* ${\left|M\right|}^{2}{\left|N\right|}^{2}={\left|N\right|}^{2}{\left|M\right|}^{2}$ *. Then*

$$\left|\left|\left|\;{\left|MN^{⁎}\right|}^{2}\right|\right|\right|\leq \frac{1}{16}\left|\left|\left|{\left(g_{2}(M^{⁎}M)+f_{1}(N^{⁎}N)\right)}^{2}\right|\right|\right|\;\left|\left|\left|{\left(g_{1}(M^{⁎}M)+f_{2}(N^{⁎}N)\right)}^{2}\right|\right|\right|.$$

Proof By the inequalities (2.16) and (2.17), we have

$$\left|\left|\left|\;{\left|MN^{⁎}\right|}^{2}\right|\right|\right|$$

$$\leq \left|\left|\left|g_{2}(M^{⁎}M)f_{1}(N^{⁎}N)\right|\right|\right|\;\left|\left|\left|f_{2}(N^{⁎}N)g_{1}(M^{⁎}M)\right|\right|\right|\leq \frac{1}{16}\left|\left|\left|{\left(g_{2}(M^{⁎}M)+f_{1}(N^{⁎}N)\right)}^{2}\right|\right|\right|\;\left|\left|\left|{\left(g_{1}(M^{⁎}M)+f_{2}(N^{⁎}N)\right)}^{2}\right|\right|\right|\;\text{(by Lemma 2.12),}$$

as required. □

Specifying Corollary 2.11 and Corollary 2.13 to a special type of functions, we get the following corollary.

Corollary 2.14 *Let* $M,N\in M_{n}$ *be such that* ${\left|M\right|}^{2}{\left|N\right|}^{2}={\left|N\right|}^{2}{\left|M\right|}^{2}$ *. Then*

$$\left|\left|\left|\;{\left|MN^{⁎}\right|}^{2}\right|\right|\right|\leq \frac{1}{4}\left|\left|\left|{\left(M^{⁎}M\right)}^{2\alpha }+{\left(N^{⁎}N\right)}^{2(1-\alpha )}\right|\right|\right|\;\left|\left|\left|{\left(M^{⁎}M\right)}^{2(1-\alpha )}+{\left(N^{⁎}N\right)}^{2\alpha }\right|\right|\right|$$

*and*

$$\left|\left|\left|\;{\left|MN^{⁎}\right|}^{2}\right|\right|\right|\leq \frac{1}{16}\left|\left|\left|{\left({\left(M^{⁎}M\right)}^{\alpha }+{\left(N^{⁎}N\right)}^{1-\alpha }\right)}^{2}\right|\right|\right|\;\left|\left|\left|{\left({\left(M^{⁎}M\right)}^{1-\alpha }+{\left(N^{⁎}N\right)}^{\alpha }\right)}^{2}\right|\right|\right|.$$

Proof The results follow by applying both of Corollary 2.11 and Corollary 2.13 to the functions $f_{2}(t)=g_{2}(t)=t^{\alpha }$ and $g_{1}(t)=f_{1}(t)=t^{1-\alpha }$. □

## CRediT authorship contribution statement

**Ahmad Al-Natoor:** Writing – review & editing.

## Declaration of Competing Interest

The authors declare that they have no known competing financial interests or personal relationships that could have appeared to influence the work reported in this paper.

## Data Availability

No data was used for the research described in the article.
